# Discrepancy between the Actions of Glucagon-like Peptide-1 Receptor Ligands in the Protection of the Heart against Ischemia Reperfusion Injury

**DOI:** 10.3390/ph15060720

**Published:** 2022-06-06

**Authors:** Ali Ismaeil, Fawzi Babiker, Suleiman Al-Sabah

**Affiliations:** 1 Department of Physiology, Faculty of Medicine, Kuwait University, P.O. Box 24923, Safat 13110, Kuwait; ali.moh.esmaeil@gmail.com; 2 Department of Pharmacology & Toxicology, Faculty of Medicine, Kuwait University, P.O. Box 24923, Safat 13110, Kuwait

**Keywords:** ischemia reperfusion, GLP-1, GLP-1 (9-36), exendin-4

## Abstract

Tirzepatide is a dual glucagon-like peptide-1 (GLP-1) and glucose-dependent insulinotropic polypeptide (GIP) receptor agonist and a promising therapy for type 2 diabetes mellitus (T2DM). GLP-1 is an incretin hormone with therapeutic potential beyond type 2 diabetes mellitus. However, GLP-1 is rapidly degraded by dipeptdyl peptidase-IV (DPP-IV) to GLP-1 (9-36). Exendin-4 (Ex-4) is a DPP-IV-resistant GLP-1 receptor agonist which, when truncated to Ex-4 (9-39), acts as a GLP-1 receptor antagonist. In the present study, hearts isolated from Wistar rats (*n* = 8 per group) were perfused with a modified Langendorff preparation. Left ventricular (LV) contractility and cardiovascular hemodynamics were evaluated by a data acquisition program and infarct size was evaluated by 2,3,5-Triphenyl-2H-tetrazolium chloride (TTC) staining and cardiac enzyme levels. Hearts were subjected to 30 min regional ischemia, produced by ligation of the left anterior descending (LAD) coronary artery followed by 30 min reperfusion. Hearts were treated during reperfusion with either the non-lipidated precursor of tirzepatide (NLT), GLP-1, GLP-1 (9-36), or Ex-4 in the presence or absence of Ex-4 (9-39). Infusion of GLP-1 (9-36) or Ex-4 protected the heart against I/R injury (*p* > 0.01) by normalizing cardiac hemodynamic and enzyme levels. Neither GLP-1, NLT, nor Ex-4 (9-39) showed any protection. Interestingly, Ex-4 (9-39) blocked Ex-4-mediated protection but not that of GLP-1 (9-36). These data suggest that Ex-4-mediated protection is GLP-1-receptor-dependent but GLP-1 (9-36)-mediated protection is not.

## 1. Introduction

Cardiovascular diseases (CVDs) are the most common cause of death worldwide [1]. Ischemic heart disease (IHD) is the leading cause of morbidity and mortality globally [2]. Myocardial reperfusion therapy is currently the only standard therapy for the protection of the ischemic heart. However, the development of complications in the first few years following infarction persists [3]. These complications include in-hospital mortality, recurrent myocardial infarction, and fatal left ventricular (LV) remodelling leading to heart failure [4]. The mechanisms responsible for ischemia/reperfusion (I/R) injury include opening of the mitochondrial permeability transition pore (mPTP), changes in intracellular pH, intracellular calcium overload, the activation of proteases, and the generation of oxidative stress by reactive oxygen species (ROS) and inflammation mediated by cytokines and complements [5,6,7]. I/R results in notable cardiomyocyte necrosis and apoptosis, which are proven causes of I/R injury [8]. Several therapeutic interventions to prevent myocardial I/R injury have been investigated. Unfortunately, the translation of such cardio-protective interventions to clinical trials has been rather disappointing [5,9]. Therefore, there is an urgent need for more effective regimens [10,11,12].

Glucagon-like peptide-1 (GLP-1) is an incretin hormone released from the gastrointestinal tract in response to feeding and functions primarily to potentiate insulin secretion from pancreatic β-cells in a glucose-dependent manner [13]. As a result, the GLP-1 receptor (GLP-1R) has become an attractive target in the treatment of type 2 diabetes mellitus (T2DM) [14,15]. Unfortunately, native GLP-1 has a very short half-life (1–2 min). The enzyme dipeptdyl peptidase-IV (DPP-IV) cleaves GLP-1 at its N-terminus to give GLP-1 (9-36) [16]. Exendin-4 (Ex-4), a peptide first discovered in the venom of the Gila monster *Heloderma suspectum*, acts as potent GLP-1R agonist. An amino acid substitution at position 2 (alanine to glycine) in the reptilian peptide results in DPP-IV-resistance and a longer circulating half-life. Removal of the first eight amino acids of Ex-4 results in Ex-4 (9-39), a GLP-1R antagonist that has been used extensively in the study of the physiology of GLP-1 [17].

GLP-1R is widely expressed and GLP-1 is now understood to be a pleiotropic hormone with therapeutic potential beyond type 2 diabetes mellitus. GLP-1R agonists have been shown to be both neuro- and cardio-protective [13]. Interestingly, the GLP-1 metabolite GLP-1 (9-36) has been shown to protect the heart against I/R reperfusion injury. Furthermore, the cardio-protective actions of GLP-1 (9-36) appear to be independent of the GLP-1R [18]. Recently, tirzepatide, a dual GLP-1R and GIP receptor agonist, is undergoing clinical trials for the treatment of type 2 diabetes [19]. This drug may have potential as a therapy for IHD. Hence, in the present study we investigated the potential role of GLP-1, Ex-4, GLP-1 (9-36), and the non-lipidated precursor of tirzepatide (NLT) in the protection of the heart against I/R injury during reperfusion to further understand the cardio-protective potential of these peptides.

## 2. Results

The end point for the evaluation of the effect of I/R and its treatments on the heart varies between laboratories. Some research groups use LV hemodynamics and contractility as an end point to evaluate the effect of I/R and its treatment on the heart muscle and its performance. Other groups use the infarct size, which is crucial for the prognosis of the disease, as an end point. Coronary and coronary vascular resistance were also used to evaluate the perfusion of the flow. In this study, for a full picture of the effect, we used LV hemodynamics, contractility, and coronary dynamics as end points. Furthermore, we used infarct size and cardiac enzyme levels as a confirmation of our findings. Heart hemodynamics during stabilization, ischemic, and reperfusion periods were evaluated. Left ventricular dynamics were determined throughout the experiment by measuring LVEDP, maximum DPmax, and LV ±dP/dt. Coronary vascular dynamics were evaluated by measuring CF and CVR. Body weights and LV weights were not significantly different between the experimental groups. The values for cardiovascular dynamics in all study groups were presented as percentages of the baseline data.

**Figure 1:**
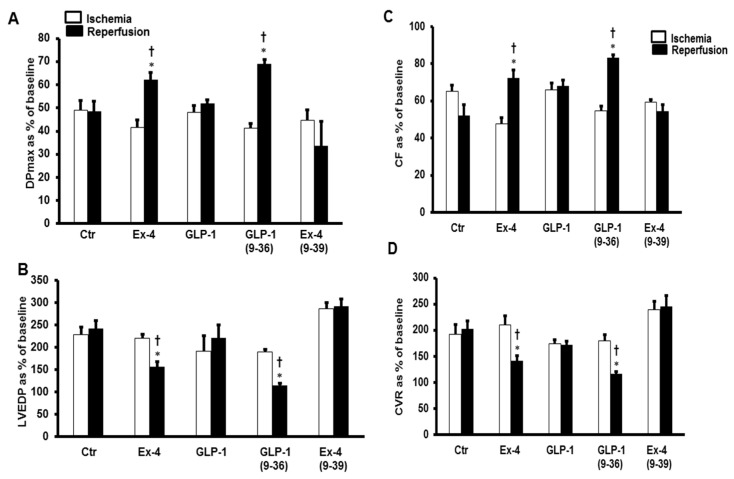
GLP-1 protection of the heart against ischemia reperfusion injury (*n* = 8 per group). Post-ischemic recovery in myocardial hemodynamics (**A**) DPmax, and (**B**) LVEDP, (**C**) CF and (**D**) CVR. The data were computed at 30 min reperfusion and are expressed as means ± SEM. Ctr, control; DPmax, maximum developed pressure; LVEDP, left ventricular end diastolic pressure; CF, coronary flow; CVR, coronary vascular resistance. * *p* < 0.001 compared to respective control. ^†^ *p* < 0.001 compared to ischemic period.

Left ventricular dynamics, contractility, and coronary vascular dynamics were not significantly different between the groups when at baseline heart perfusion. Ischemia, which followed the baseline perfusion, resulted in a considerable deterioration in the heart functions which was reversed by GLP-1 (9-36) and Ex-4 but not by GLP-1 (Figure 1).

**Figure 2:**
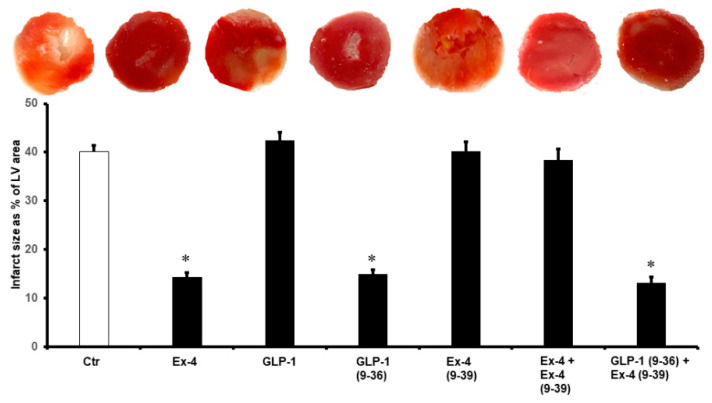
Post-ischemic infarct size in different treatment groups. Post-infarct size is significantly reduced with the introduction of Ex-4, GLP-1(9-36), and GLP-1 (9-36) + Ex-4 (9-39). Ctr, control; GLP-1, glucagon-like peptide-1; GLP-1 (9-36), glucagon-like peptide-1 (9-36); Ex-4 (9-39), exendin-4 (9-39); Ex-4, exendin-4. * *p* < 0.01 compared to the control group.

The same effect was observed in the LV ±dP/dt, which was significantly improved by the infusion of GLP-1 (9-36) and Ex-4 (Table 1). These results were confirmed by the decrease in the infarct size (Figure 2) and the levels of cardiac enzyme release, which were decreased in the presence of GLP-1 (9-36) and Ex-4 (Table 2).

**Figure 3:**
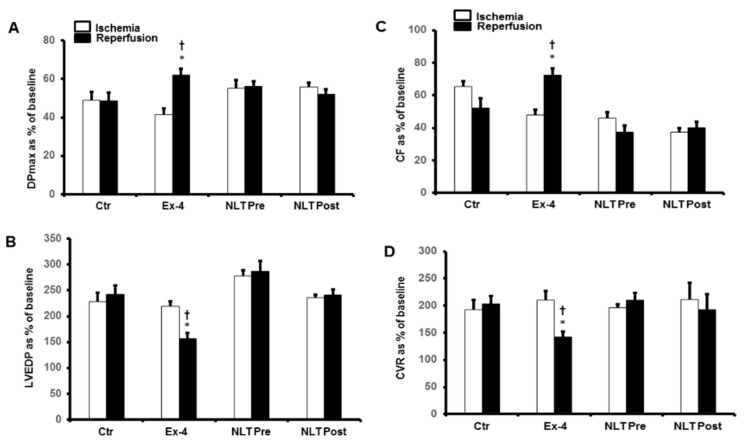
Post-ischemic recovery of CVR in the presence of exendin-4 and NLT infused before ischemia or at reperfusion. (**A**) DPmax, and (**B**) LVEDP, (**C**) CF and (**D**) CVR. No significant improvement in CVR was appreciated in the displayed treatment groups. The data were collected after 25 min of ischemia and 30 min of reperfusion and are presented as means ± SE. CVR, coronary vascular resistance; Ex-4, exendin-4; NLT Pre, non-lipidated tirzepatide pre-ischemia; NLT Post, non-lipidated tirzepatide post-ischemia, * *p* < 0.05 compared to the control group. ^†^ *p* < 0.05 compared to the respective ischemic period.

**Figure 4:**
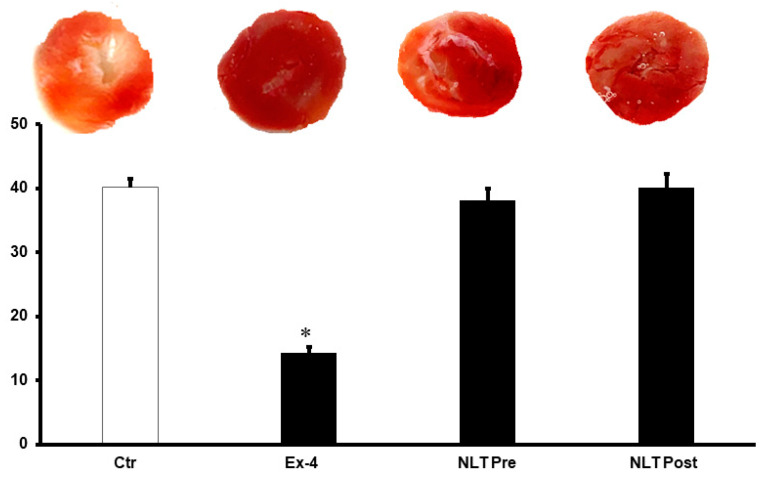
Post-ischemic infarct size in different treatment groups. Post-ischemic infarct size was not reduced by the introduction of NLT pre- and post-ischemia. Ctr, control; Ex-4, exendin-4; NLT Pre, non-lipidated tirzepatide pre-ischemia; NLT Post, non-lipidated tirzepatide post-ischemia. * *p* < 0.01 compared to the control group.

We then used NLT, the non-lipidated precursor of tirzepatide, to investigate any cross-talk between the GLP-1 and GIP receptors or possible synergy in their protection. Surprisingly, the infusion of NLT before ischemia or reperfusion did not protect the heart against I/R (Figure 3A–D). These results were confirmed by a lack of decrease in the infarct size (Figure 4) and increased cardiac enzyme levels (Table 2).

**Figure 5:**
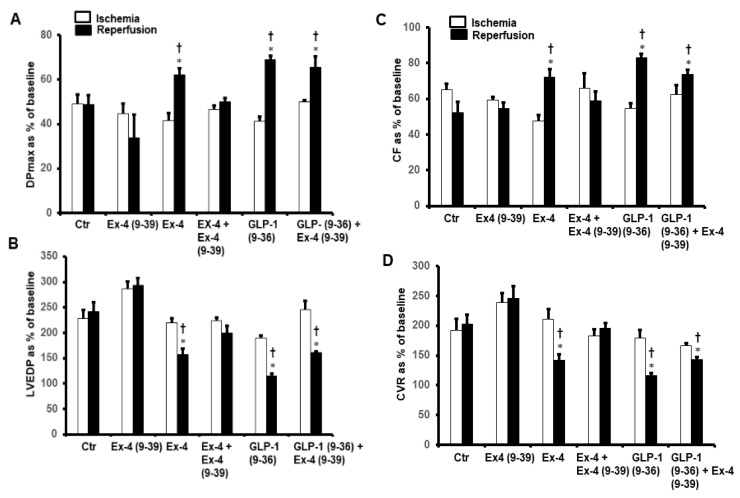
Post-ischemic recovery of CVR in the presence of exendin-4 + exendin-4 (9-39) and GLP-1(9-36) + exendin-4 (9-39). (**A**) DPmax, and (**B**) LVEDP, (**C**) CF and (**D**) CVR. The data were collected after 25 min of ischemia and 30 min of repefusion and are presented as means ± SE. LVEDP, left ventricular end diastolic pressure; DPmax, maximum developed pressure; Ex-4, exendin-4; GLP-1 (9-36), glucagon-like peptide-1 (9-36); Ex-4 (9-39), exendin-4 (9-39). * *p* < 0.05 compared to the control group; ^†^ *p* < 0.05 compared to the respective ischemic period.

To investigate whether the improvements in the hemodynamics, infarct size, and cardiac enzyme levels reported above were modulated via a signaling pathway involving GLP-1R, the GLP-1R antagonist Ex-4 (9-39) was used at reperfusion in the presence of GLP-1 (9-36) and Ex-4. Ex-4 (9-39) completely blocked the protection given by Ex-4 but not the protection produced by GLP-1 (9-36) (Figure 5A–D and Table 1 and Table 2). 

**Figure 6:**
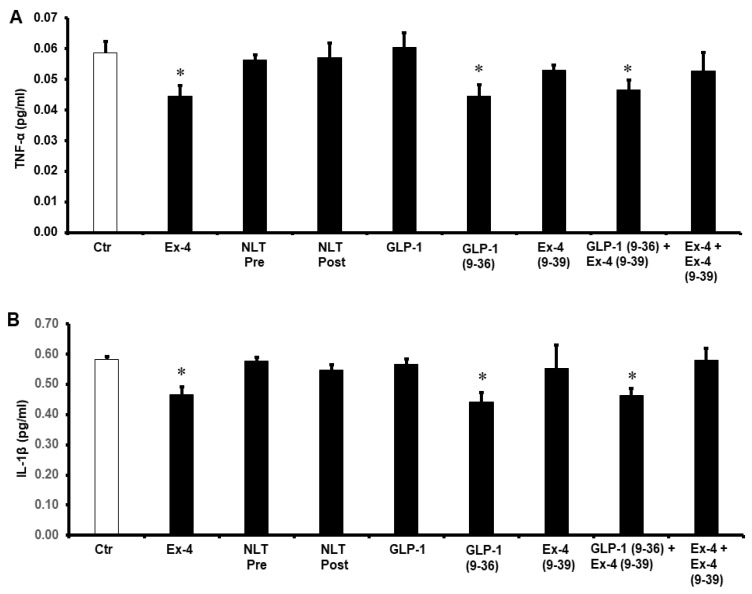

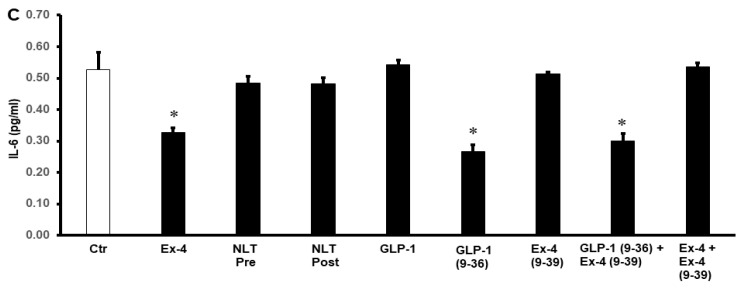
Post-ischemic reduction of (**A**) TNF-α, (**B**) IL-1β, and (**C**) IL-6 levels. Ctr, control; GLP-1, glucagon-like peptide-1; GLP-1 (9-36), glucagon-like peptide-1 (9-36); Ex-4 (9-39), exendin-4 (9-39); Ex-4, exendin-4; NLT Pre, non-lipidated tirzepatide pre-ischemia; NLT Post, non-lipidated tirzepatide post-ischemia. * *p* < 0.01 compared to the control group.

To study the possible effects of GLP-1 and NLT on the modulation of cytokines during I/R injury, the levels of TNF-α, IL-1β, and IL-6 were measured in the cardiomyocyte lysate by ELISA. Exposure of the heart to GLP-1 (9-36) and Ex-4 resulted in a significant decrease in the protein levels of TNF-α, IL-1β, and IL-6 compared to those of untreated controls (*p* < 0.05) (Figure 6). This effect was not observed in the treatment with NLT and was completely negated by Ex-4 (9-39) in the presence of Ex-4 but not when co-administered in presence of GLP-1 (9-36) (Figure 6A–C).

**Figure 7:**
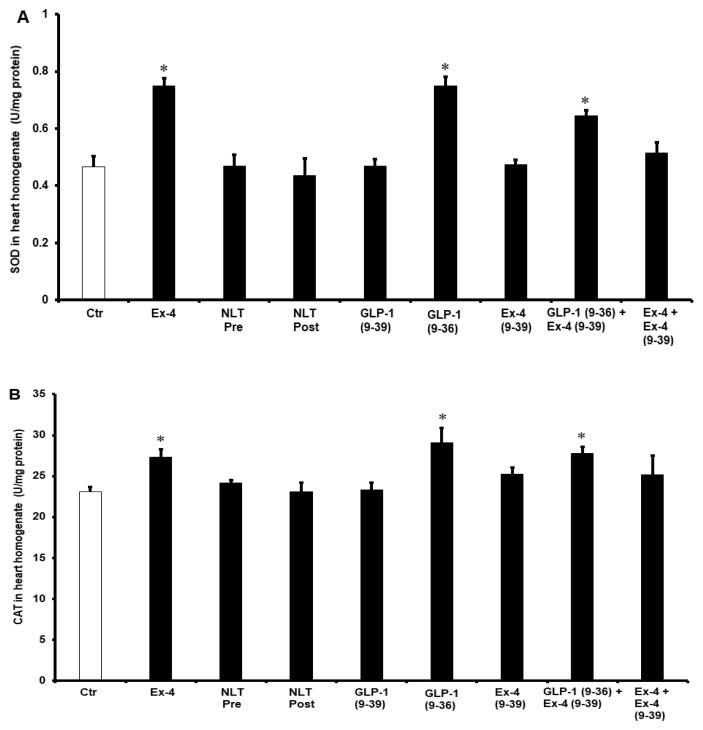
Post-ischemic recovery of (**A**) CAT and (**B**) SOD activity. Ctr, control; GLP-1 (9-36), glucagon-like peptide-1 (9-36); Ex-4 (9-39), exendin-4 (9-39); Ex-4, exendin-4; NLT Pre, non-lipidated tirzepatide pre-ischemia; NLT Post, non-lipidated tirzepatide post-ischemia. * *p* < 0.01 compared to the control group.

To understand the potential pathways of the GLP-1 protection of the heart, the antioxidant enzymes SOD and CAT and were evaluated. Treatment with GLP-1 (9-36) and Ex-4 significantly (*p* < 0.01) increased SOD and CAT levels compared to controls. This was not seen when the heart was treated with NLT and was completely abrogated by Ex-4 (9-39) in the presence of Ex-4 but not when co-administered with GLP-1 (9-36) (Figure 7A,B). 

## 3. Discussion

There are currently several GLP-1 receptor agonists used clinically for the treatment of both type 2 diabetes mellitus and obesity, for example, liraglutide, lixsenatide, semaglutide, dulaglutide and exenatide [13]. This class of medication has also been shown to display cardiovascular protection in high-risk patients with T2DM [20]. It is thought that GLP-1R agonists’ cardiovascular effects are at least partially independent of their ability to control glycemia and body weight [21,22]. GLP-1R is expressed in the heart, although its exact location is unclear; therefore, it is possible that GLP-1R agonists exert direct, as well as indirect, effects upon the heart [23,24]. Initially thought of as simply the degradation product of GLP-1, GLP-1 (9-36) has since been shown to have distinct physiological effects compared to its parent peptide [25]. Furthermore, GLP-1 (9-36) has been shown to be cardio-protective in a murine model of ischemia-reperfusion injury. Intriguingly, GLP-1 (9-36)’s cardio-protective properties were preserved in GLP-1 receptor knockout cardiomyocytes, suggesting GLP-1R-independent activity [18].

The present study also demonstrates the protective effects of GLP-1 (9-36) and Ex-4 on the heart against I/R injury. This involved the GLP-1R for Ex-4 but not for GLP-1 (9-36). Both Ex-4 and GLP-1 (9-36) have previously been shown to have cardio-protective effects [18,26,27]. However, the precise mechanism and the involvement of GLP-1R in the protection of the heart against I/R injury has yet to be determined. Furthermore, evidence for the potential protection of these peptides when infused at reperfusion, which is clinically more relevant than infusion before ischemia, is also lacking.

No protection of the heart against I/R injury by native GLP-1 could be demonstrated in the present study. This contrasts with previous studies which report GLP-1-mediated protection of the heart against I/R injury in both animal models of diabetes and diabetic patients [28,29]. The discrepancy between these studies and the results reported here could be due to GLP-1 protecting the diabetic heart under conditions of hyperglycemia or organ remodeling created by the presence of diabetes. The animal model, the dose and the time of infusion could all contribute to the reported discrepancies. A direct effect for GLP-1 in non-diabetic animals has also been previously reported; however, in these studies, GLP-1 was infused before the ischemic insult. These results can be explained by GLP-1 being protective to the heart against I/R injury in diabetic models or when infused before ischemia. The protocol used in the present study infuses GLP-1 at reperfusion, which is clinically more relevant than perfusion before ischemia. 

Intriguingly, the present study demonstrates that the GLP-1 degradation product GLP-1 (9-36) displays GLP-1R-independent protection of the heart against I/R injury. As mentioned previously, GLP-1 (9-36) has been reported to be both an antagonist and a weak partial agonist of the GLP-1R as well as being biologically inactive [30,31,32]. Furthermore, Sauvé et al. demonstrated that genetic deletion and pharmacological inhibition of DPP IV, which inhibits the production of GLP-1 (9-36), improved cardiovascular outcomes following myocardial infarction in a murine model [33]. In contrast, other studies have shown GLP-1 (9-36) to be protective against I/R injury in various tissues and organs [24,34,35,36]. To our knowledge, the present study is the first to demonstrate that GLP-1 (9-36) is protective against I/R injury when infused at reperfusion.

**Figure 8:**
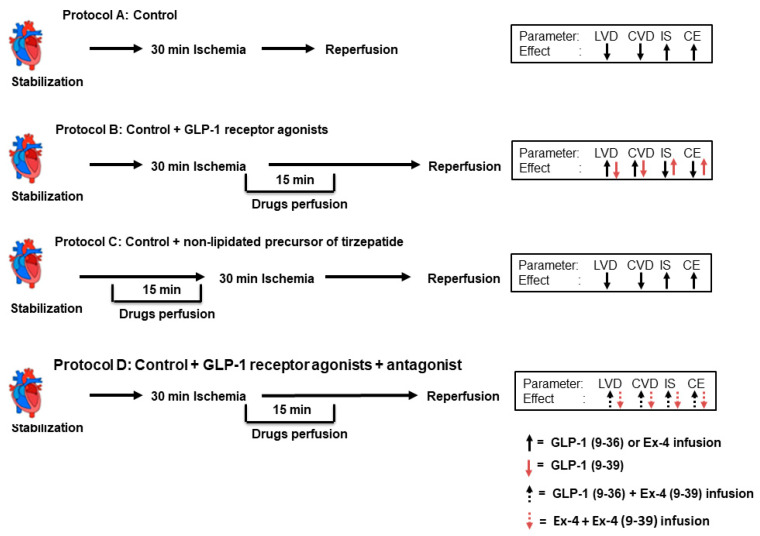
Schematic representation showing the experimental protocols used for the study (*n* = 8 per group). (**A**) Unprotected ischemia-reperfusion (control). (**B**) Infusion with GLP-1, GLP-1 (9-36), and Ex-4 (**C**) Infusion of non-lipidated precursor of tirzepatide. (**D**) Infusion with GLP-1, GLP-1 (9-36), and Ex-4 in presence of Ex-4 (9-39).

Treatment of isolated hearts with either Ex-4 or GLP-1 (9-36) at reperfusion protected the heart against I/R injury and normalized LV dynamics and coronary vascular dynamics to those of controls. Whereas the cardio-protective effects of Ex-4 were inhibited by infusion of the GLP-1R antagonist Ex-4 (9-39), this was not the case for GLP-1 (9-36) (Figure 8). These data suggest that the cardio-protective effects of Ex-4 require the GLP-1R, which agrees with previous studies which demonstrate the involvement the GLP-1R in the protection of the heart [24,33]. Our data are also in agreement with other studies which demonstrate a GLP-1R-independent mechanism for GLP-1 (9-36) in the protection of the heart [24]. Although Ex-4 has been shown to be protective of the heart before ischemia, its infusion at reperfusion did not show protection in previous studies [22,29]. This contrasts with the data presented in the present study. This discrepancy may be due to either differences in the animal models or the procedures employed. The results of the study indicate acute protection of the heart against I/R injury by Ex-4 and GLP-1 (9-36) when infused at reperfusion by a GLP-1R mechanism for Ex-4 but not for GLP-1 (9-36). One of the strengths of this ex vivo preparation is that it shows the possible separation of the direct effects of the GLP-1 metabolites on the heart from the additional insulinotropic effects of GLP-1 that occur in vivo. 

In addition, we used NLT in this study to investigate potential improvement in the protective effects compared to Ex-4 and GLP-1 (9-36). Interestingly, infusion with NLT before ischemia or at reperfusion did not protect the heart against I/R injury. NLT was proven to be effective as a therapy for type 2 diabetes, but its effect on the protection of the heart against I/R injury has not yet been reported [19]. Although NLT is a agonist at the GLP-1R, which plays a protective role in I/R [37], it does not show protection of the heart similar to the other agonists of the GLP-1R [38,39]. However, contrasting results were also reported [40,41]. The masking of the protective effect of NLT could be mainly due to its agonism of GIPR, which was reported to exacerbate ischemic injury, and its blockade is beneficial to the ischemic heart [40].

This study reported an anti-inflammatory effect for Ex-4 and GLP-1 (9-36), as treatment of the heart with these compounds resulted in a significant reduction in TNF-α, IL-1β, and IL-6. Although the presence of immune cells in the isolated heart was unlikely, several studies have shown that I/R causes its damage and cardiac dysfunction to the heart partly by promoting pro-inflammatory cytokine formation [12,42]. Treatment of the rat heart with Ex-4 and GLP-1 (9-36) showed a significant reduction in these pro-inflammatory cytokine protein levels. GLP-1R agonists were reported to have anti-inflammatory properties, which might explain their protective effects in this study [43]. These agonists showed a significant reduction in TNF-α, IL-1β, and IL-6 [44,45]. Lowering the TNF-α [46], IL-1β [47,48], and IL-6 [47] protein levels was proven to be essential in the protection of the heart against I/R injury and was shown to decrease apoptosis in cardiac myocytes [47,48]. The results of this study are consistent with the above findings.

In this study, we reported an increase in the levels of the antioxidant enzymes SOD and CAT after the treatment with Ex-4 and GLP-1 (9-36), which proves the presence of antioxidant activity in these compounds. Although ROS are essential for many beneficial physiological processes, their contribution to pathological conditions is indispensable [49], and GLP-1R was proven to be a strong antioxidant [50]. Ex-4 [51] and (GLP- (9-36) [52] were reported to be essential effectors in the reduction of ROS levels in the context of hyperglycemia. Reductions in ROS by Ex-4 and GLP-1 (9-36) in the protection of cardiomyocytes were also reported previously [53,54]. Nevertheless, it was in a pretreatment procedure, where Ex-4 and GLP-1 (9-36) protected the heart against hypoxic damage [55]. Similar protective effects by decreasing ROS levels were reported previously by our laboratory [56,57]. We have also previously reported increased levels of SOD and CAT in other procedures that showed protection of the heart against I/R injury [46]. 

## 4. Materials and Methods

### 4.1. Ischemia Reperfusion Study

Age-matched male Wistar rats weighing between 270 and 300 g (*n* = 8 per group) were used in this study. Animal treatment and handling were carried out according to the guidelines of Animal Resource Center of Kuwait University, Kuwait. Rats were maintained at 22 °C on 12 h light/dark cycle (7 am–7 pm), and water and food were unrestrictedly available. Rats were anesthetized with intraperitoneal injections of sodium pentobarbital (60 mg/kg), and heparin (1000 U/kg) was administered through the femoral vein. Heart cannulation and perfusion were performed as described previously [10,58]. Briefly, isolated heart was retrograde perfused with a freshly prepared Krebs–Hensleit buffer, pH 7.35 to 7.45 at 37.0 ± 0.5 °C and was supplied with CO_2_ (5%) and O_2_ (95%). Heart instrumentation included placing pacing electrodes on the right atrium (RA) appendage to maintain physiological heartbeat. Regional ischemia was induced by occluding left anterior descending (LAD) coronary artery for 30 min. The LAD was encircled by a snare at 0.5 cm below the atrioventricular groove, and a small, rigid plastic tube was positioned between the heart and the snare to ensure complete occlusion of the coronary artery. Subsequently, the heart was reperfused for 30 min. The flow of the perfusate was measured downstream of the aortic cannula using a “Statham pressure transducer” (P23 Db). Preload was held constant at 6 mmHg under basal controlled conditions, and the perfusion pressure (PP) was held constant at 50 mmHg throughout the experimental procedure in all protocols. Constant PP was ensured electronically by means of the perfusion assembly (“Module PPCM type 671 (Hugo Sachs Elektronik-Harvard Apparatus GmbH, March, Germany”)), an effective system for the accurate adjustment of PP between 5 mmHg and 150 mmHg with ±1 mmHg accuracy level.

### 4.2. Study Protocol and Study Groups

A total of 72 hearts were subdivided into 9 groups (*n* = 8 per group) and subjected to four different experimental protocols. The control group was subjected to ischemia and reperfusion without any other treatment; the second group was treated with NLT (0.1 µM) started 15 min before and continued for 15 min; the third to seventh groups were subjected to control procedures with infusion of (0.1 µM each) Ex-4, NLT, GLP-1 (9-39), GLP-1 (9-36), and Ex-4 (9-39) 5 min before the start of reperfusion and continued for 10 min during reperfusion; and the eighth and ninth groups were treated with GLP-1 (9-36) or Ex-4 in presence of the GLP-1R antagonist Ex-4 (9-39) (0.1 µM), as summarized in Figure 1. The antagonists were always infused 5 min before the infusion of the drug in question. Animals were randomly assigned to groups and the treatments were applied randomly. All hearts were subjected to 30 min of ischemia produced by LAD coronary artery occlusion. Hearts were then reperfused for 30 min (Figure 8). 

### 4.3. Data Collection and Processing

The function of LV was evaluated by the assessment of LV end diastolic pressure (LVEDP), LV developed pressure (DPmax), and LV contractility (±dP/dt). The coronary-vascular dynamics were evaluated by the assessment of coronary vascular resistance (CVR) and coronary flow (CF). Cardiovascular functions were measured as described in the previous studies [56,59]. Briefly, a water-filled latex balloon was placed and secured in LV cavity. The balloon was attached to a pressure transducer and a “DC-Bridge amplifier (DC-BA)” of the pressure module (DC-BA type 660, Hugo-Sachs Electronik, March, Germany) and interfaced with a personal computer for online monitoring of DPmax. Left ventricular developed pressure was derived from online acquisition of “LVESP using Max-Min module (Number MMM type 668” (Hugo Sachs Elektronik-Harvard ApparatusGmbH, March, Germany) which could convert the output from DC bridge amplifier to DPmax by subtracting LVEDP from the LVEDP. 

Coronary flow was continuously measured using an electromagnetic flow probe interfaced with a personal computer and attached to the inflow of the aortic cannula. The continuous monitoring of the coronary flow in ml/min was performed digitally using suitable software and was verified manually by timed collection of coronary effluent. The CVR and hemodynamics data were determined every 10 s using an “online data acquisition program” (Isoheart software V 1.524-S, Hugo-Sachs Electronik, March, Germany). By the end of each experiment, hearts were snap-frozen in liquid nitrogen, then stored in −80 °C for further analysis. 

### 4.4. Sample Collection and Storage

Coronary effluent was collected manually at the apex of the heart from the coronary outflow in small tubes at the end of the reperfusion phase. The hearts were also collected at the end of reperfusion. All heart samples and coronary effluents were frozen in liquid nitrogen and stored at −80 °C for further analysis.

### 4.5. Infarct-Size Evaluation 

Hearts were collected after 30 min of reperfusion and stored overnight at −20 °C. The next day, the hearts were sliced into 4–5 pieces from apex to base. The slices were then incubated in 1% 2,3,5-Triphenyl-2H-tetrazolium chloride (TTC) solution in isotonic (pH 7.4) phosphate buffer and then fixed in 4% formaldehyde. The red and pale non-stained areas of every slice were indicated manually on the image. The infarct size as a percentage of the LV was calculated for every heart. Quantification of the LV and infarct size was performed using ImageJ (Image J, Wayne Rasband and National Institute of Health, Bethesda, MD, USA). Cardiomyocyte injury was confirmed by measuring creatine kinase (CK) and lactate dehydrogenase (LDH) release in the coronary effluent during the reperfusion period, as described previously by Ferrera et al. [60]. 

### 4.6. Protein Extraction from the Hearts

At the end of the experiments, the hearts were snap-frozen in liquid nitrogen and subsequently stored at −80 °C for further analysis. Left ventricle tissue was homogenized in a lysis buffer (MOPS, 20 mM; KCl, 150 mM; Mg Acetate, 4.5 mM; Triton X, 1%) containing protease inhibitor cocktail tablet (Roche Applied Science, Mannheim, Germany, Cat#05892970001) and centrifuged at 12,000× *g* for 15 min at 4 °C. The supernatant containing the proteins was then collected, aliquoted, and stored for further analysis. Protein levels were assayed using bicinchoninic acid protein assay (Pierce Chemical Co., Rockford, IL, USA, Cat# 23227). 

### 4.7. Antioxidant Enzyme Superoxide Dismutase Status

Superoxide dismutase (SOD; EC.1.15.1.1) and Catalase (CAT; EC.1.11.1.6) activity were measured in cardiomyocyte lysate (*n* = 4) by UV spectrophotometer activity with RANDOX kits (Cat. No. SD 125; Randox Labs Ltd., Crumlin, UK) according to the manufacturer’s instructions. 

### 4.8. Estimation of Inflammatory Cytokines 

The LV homogenate (*n* = 4) was used to evaluate the expression of the proinflammatory cytokines TNF-α (cat# MBS175904), IL-1 (cat# MBS825017), and IL-6 (cat# MBS355410) using enzyme-linked immunosorbent assay (ELISA). The protein content was measured in the samples, which were diluted as suggested by the manufacturer’s assay protocols for the respective commercial kits (Biosource International, Camarillo, California, USA).

### 4.9. Data Analysis 

The data were expressed as the means ± SE. Two-way analysis of variance (ANOVA) was used for repeated measures within each group and between the groups. In the case where analysis of variance illustrated a significant difference, the post hoc analysis with Tukey test was used for further comparison. Infarct size was analyzed using student *t*-test. Comparisons were made between group means and their respective controls. Values of *p* < 0.05 were considered statistically significant for the analysis.

## 5. Conclusions

**Figure 9:**
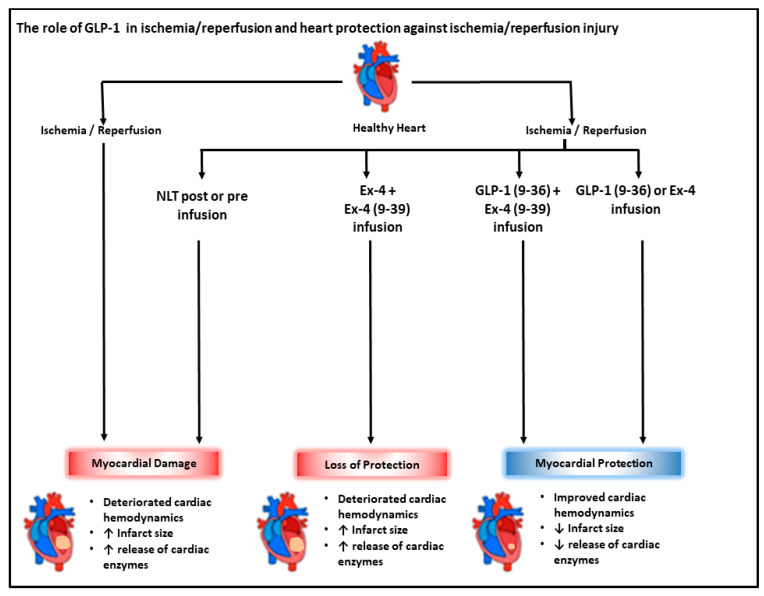
Schematic representation showing the interactions between I/R injury and GLP-1 related peptides. Ischemia/reperfusion-induced cardiac injury characterized by impaired cardiac hemodynamics and enhanced release of cardiac enzymes, LDH, and CK. GLP-1 (9-36) and Ex-4 treatment improved cardiac hemodynamic and attenuated release of cardiac enzymes. Ex-4 protection involved downstream signaling via the GLP-1 receptor; however, the protection elicited by GLP-1(9-36) appears to be GLP-1-receptor independent.

The data presented demonstrate that Ex-4-mediated protection of the heart against I/R injury when infused at reperfusion is GLP-1-receptor-dependent. In contrast, GLP-1 (9-36) protection under the same conditions appears to be GLP-1-receptor-independent. These findings highlight the need for a greater understanding of the mechanisms of GLP-1 (9-36)’s GLP-1-receptor independence with the aim of translating these findings to the clinic (Figure 9).

## Figures and Tables

**Table 1:** The effect of GLP-1 receptor ligands on heart contractility in presence or absence of the GLP-1R antagonist Ex-4 (9-39).

Treatment	+dP/dt		−dP/dt	
	Ischemia	Reperfusion	Ischemia	Reperfusion
Ctr	57.30 ± 4.81	55.62 ± 5.05	55.41 ± 3.68	48.27 ± 4.03
Ctr + EX-4	46.65 ± 1.38	73.53 ± 3.20 * ^†^	42.73 ± 1.83	71.36 ± 3.61 * ^†^
Ctr + NLT Rep	52.90 ± 4.82	48.55 ± 2.22	51.16 ± 2.66	44.17 ± 1.27
Ctr + NLT pre	54.18 ± 3.75	44.90 ± 5.41	56.94 ± 4.11	54.65 ± 4.71
Ctr + GLP-1	46.63 ± 2.44	51.13 ± 2.96	44.90 ± 1.66	51.68 ± 3.71
Ctr + GLP-1 (9-36)	48.33 ± 4.12	71.28 ± 1.98 * ^†^	48.10 ± 2.79	66.11 ± 1.91 * ^†^
Ctr + Ex-4 (9-39)	65.56 ± 5.36	42.22 ± 7.56	59.58 ± 2.04	51.10 ± 2.83
Ctr + Ex-4 + Ex-4 (9-39)	63.44 ± 5.10	68.32 ± 4.61	51.22 ± 6.05	55.13 ± 8.33
Ctr + GLP-1 (9-36) + Ex-4 (9-39)	60.86 ± 3.60	76.04 ± 1.88 * ^†^	59.02 ± 3.52	73.63 ± 2.32 * ^†^

Ctr, control; DPmax, maximum developed pressure; LVEDP, left ventricular end diastolic pressure; CF, coronary flow; CVR, coronary vascular resistance. * *p* < 0.001 compared to respective control. ^†^ *p* < 0.001 compared to ischemic period.

**Table 2:** Effects of GLP-1 isoforms on the heart contractility in presence or absence of GLP-1R antagonists EX-(9-39) on the cardiac enzyme levels.

Treatment	CK (IU/L)	*p* Value	LDH (IU/L)	*p* Value
Ctr	15.78 ± 0.46	-	10.95 ± 1.02	-
Ctr + EX-4	10.73 ± 0.56 * ^†^	0.001	7.45 ± 0.47 * ^†^	0.01
Ctr + NLT Rep	10.11 ± 0.46	0.369	10.40 ± 0.17	0.558
Ctr + NLT pre	14.95 ± 0.84	0.448	15.02 ± 0.76	0.471
Ctr + GLP-1	13.78 ± 1.40	0.215	9.43 ± 0.52	0.131
Ctr + GLP-1 (9-36)	10.58 ± 0.85 * ^†^	0.001	6.25 ± 0.82 * ^†^	0.01
Ctr + Ex-4 (9-39)	15.33 ± 0.95	0.635	10.88 ± 0.37	0.937
Ctr + Ex-4 + Ex-4 (9-39)	14.70 ± 1.59	0.427	10.33 ± 0.44	0.487
Ctr + GLP-1 (9-36) + Ex-4 (9-39)	10.48 ± 0.78 * ^†^	0.01	6.28 ± 0.49 * ^†^	0.01

CK, creatine kinase; LDH, lactate dehydrogenase; Ctr, control; DPmax, maximum developed pressure; LVEDP, left ventricular end diastolic pressure; CF, coronary flow; CVR, coronary vascular resistance. * *p* < 0.001 compared to respective control. ^†^ *p* < 0.001 compared to ischemic period.

## Data Availability

Data is contained within the article.
